# The association of fatigue, pain, depression and anxiety with work and activity impairment in immune mediated inflammatory diseases

**DOI:** 10.1371/journal.pone.0198975

**Published:** 2018-06-07

**Authors:** Murray W. Enns, Charles N. Bernstein, Kristine Kroeker, Lesley Graff, John R. Walker, Lisa M. Lix, Carol A. Hitchon, Renée El-Gabalawy, John D. Fisk, Ruth Ann Marrie, on behalf of the CIHR Team in Defining the Burden and Managing the Effects of Psychiatric Comorbidity in Chronic Immunoinflammatory Disease

**Affiliations:** 1 Department of Psychiatry, Max Rady College of Medicine, Rady Faculty of Health Sciences, University of Manitoba, Winnipeg, Manitoba, Canada; 2 Department of Community Health Sciences, Max Rady College of Medicine, Rady Faculty of Health Sciences, University of Manitoba, Winnipeg, Manitoba, Canada; 3 Department of Internal Medicine, Max Rady College of Medicine, Rady Faculty of Health Sciences, University of Manitoba, Winnipeg, Manitoba, Canada; 4 George & Fay Yee Centre for Healthcare Innovation, Winnipeg, Manitoba, Canada; 5 Department of Clinical Health Psychology, Max Rady College of Medicine, Rady Faculty of Health Sciences, University of Manitoba, Winnipeg, Manitoba, Canada; 6 Department of Anesthesia and Perioperative Medicine, Max Rady College of Medicine, Rady Faculty of Health Sciences, University of Manitoba, Winnipeg, Manitoba, Canada; 7 Department of Psychiatry, Faculty of Medicine, Dalhousie University, Halifax, Nova Scotia, Canada; University of Birmingham, UNITED KINGDOM

## Abstract

Impairment in work function is a frequent outcome in patients with chronic conditions such as immune-mediated inflammatory diseases (IMID), depression and anxiety disorders. The personal and economic costs of work impairment in these disorders are immense. Symptoms of pain, fatigue, depression and anxiety are potentially remediable forms of distress that may contribute to work impairment in chronic health conditions such as IMID. The present study evaluated the association between pain [Medical Outcomes Study Pain Effects Scale], fatigue [Daily Fatigue Impact Scale], depression and anxiety [Hospital Anxiety and Depression Scale] and work impairment [Work Productivity and Activity Impairment Scale] in four patient populations: multiple sclerosis (n = 255), inflammatory bowel disease (n = 248, rheumatoid arthritis (n = 154) and a depression and anxiety group (n = 307), using quantile regression, controlling for the effects of sociodemographic factors, physical disability, and cognitive deficits. Each of pain, depression symptoms, anxiety symptoms, and fatigue individually showed significant associations with work absenteeism, presenteeism, and general activity impairment (quantile regression standardized estimates ranging from 0.3 to 1.0). When the distress variables were entered concurrently into the regression models, fatigue was a significant predictor of work and activity impairment in all models (quantile regression standardized estimates ranging from 0.2 to 0.5). These findings have important clinical implications for understanding the determinants of work impairment and for improving work-related outcomes in chronic disease.

## Introduction

Immune-mediated inflammatory diseases (IMIDs) including inflammatory bowel disease (IBD), multiple sclerosis (MS) and rheumatoid arthritis (RA) have a substantial societal burden, in addition to the associated personal cost and challenge, owing to their increasing prevalence, and the impacts of chronicity, acute exacerbations and, for some, progressive disability [1–4]. A substantial part of the burden of illness in these conditions relates to functional impairment, particularly the affecting the ability to work [5–7]. IBD, MS, and RA patients are all at increased risk of leaving the workforce early due to their disease [8–10]. Each of these IMIDs affect different organ systems , and has unique manifestations that contribute to functional impairment such as impairment of mobility and upper/lower limb function (MS and RA) and impairment of cognition (MS). However, IMIDs also share some common symptomatic features that may be responsible for functional impairment including fatigue and pain (e.g. IBD—abdominal pain; MS—neuropathic pain, spasticity; RA—joint pain) [11–13].

People with IMIDs have a high prevalence of psychiatric comorbidity, though it is not clear if the mechanism relates to the immune-inflammatory state or sequelae of the chronic illness [14–16]. For example, patients with IBD have markedly higher rates of psychiatric disorders than the general population, with more than 25% having a lifetime diagnosis of depression and 30% an anxiety disorder [17,18]. Anxiety and depressive disorders have also been commonly associated with MS; the lifetime prevalence of depression in MS is as high as 50% [19–21]. Patients with RA also have a high prevalence of psychiatric comorbidity; the reported lifetime prevalence of depression is 41–66% and the lifetime prevalence of anxiety disorders is as high as 70% [22–24].

Psychiatric disorders have themselves been identified as major causes of disease burden and disability worldwide [25], and it would therefore be reasonable to predict that comorbid psychiatric symptoms of depression and anxiety in patients with IMID would be associated with increased impairment. A large community survey found that in several chronic disease conditions, which included hypertension, arthritis, asthma and ulcers, the associated role impairment was almost entirely explained on the basis of concurrent mental disorders [26].

The main source of financial security for most people is their ability to engage in competitive employment. Chronic health problems may create limitations in the ability to work and this in turn has a negative impact on the person, the employer and the economy in general. This is one of the key costs of chronic illness. Loss of productivity may be seen in three ways: a) absence from work, b) limitation in productivity while at work, also known as presenteeism [27], and c) loss of ability to work.

In the case of IMIDs, several studies of individual IMIDs have suggested that psychiatric comorbidity such as depression and anxiety may also be important determinants of work impairment. In separate studies, depression has been associated with reduced employment functioning [28] and presenteeism [29] in MS. In RA, depression has been found to predict future disability pension [30] and self-reported functional impairment [31]. Significantly disabled patients with IBD are more likely to have a lifetime history of major depression [32], and in another study, depression and anxiety symptoms were associated with being unemployed in IBD [33].

Recent research findings in individuals with distress disorders such as major depression and anxiety disorders suggest an important role of inflammation in the pathophysiology of these conditions, even in the absence of known IMIDs [34–37]. Inflammatory biomarkers are elevated in many patients with major depressive disorder and anxiety disorders [35]. Anti-inflammatory treatments for mood disorders have shown preliminary evidence of efficacy in reducing depression and mania symptoms [36]. Based on the accumulated clinical and pre-clinical evidence, a distinct inflammatory subtype of depression has been proposed [37]. These findings suggest a further rationale for considering and studying the role of depression and anxiety in individuals with IMIDs.

Although anxiety and depressive disorders on their own have clearly been implicated as causes of work impairment, the impact of the comorbidity of IMID and mental health conditions has not been well-studied. The foregoing review suggests that four types of distressing symptoms, namely pain, fatigue, depression, and anxiety may be broadly important in determining work impairment across a range of IMIDs. Much of the research on work impairment in IMIDs has considered disease conditions individually and not collectively, and as such, studies to date do not directly address common causes of work disability across inflammatory diseases. Further, most of the studies to date have focused on one narrow or specific indicator of work disability, such as employment status or disability pension rather than taking a broader view of employment functioning in terms of work absenteeism, presenteeism, and general activity impairment. Finally, prior work has been largely limited to smaller clinical samples.

The present study sought to evaluate the relationship between four potentially broadly relevant distress symptoms (pain, fatigue, depression and anxiety) and work impairment (including absenteeism, presenteeism and general activity impairment) in cohorts of patients with MS, RA, IBD and depression/anxiety disorders, while controlling for the impact of potentially significant factors such as sociodemographic variables, physical impairment, cognitive function and other concurrent health conditions. A mixed group of individuals with depression and anxiety disorders was included in the present study because depression and anxiety disorders are all common, they are very frequently comorbid, they have numerous shared symptomatic manifestations, and because the aim of the study was to examine dimensional measures of both depression and anxiety symptoms as correlates of functional impairment.

## Methods

### Participants

Participants in the present cross-sectional study were recruited from clinical populations and research registries maintained in Manitoba. This included persons with IBD–Crohn’s disease or ulcerative colitis–[38], N = 247, persons with MS–Poser [39] or revised McDonald [40] criteria—N = 255, and persons with RA [41], N = 154,. Persons with IBD, MS, and RA were considered to have immune-mediated inflammatory diseases (IMID). Persons with Diagnostic and Statistical Manual of Mental Disorders Fourth Edition [42] major depressive disorder or any DSM-IV anxiety disorders, N = 308, were recruited from psychology, psychiatry and primary care practices. Participant recruitment took place between November 2014 and July 2016.

Inclusion criteria across groups included: age 18 years or older, able to provide informed consent, adequate knowledge of English language to complete questionnaires, and ability to commit to planned longitudinal participation for three years. (However, the present study used data from thebaseline assessment only). The presence of an IMID was an exclusion criterion for the anxiety/depression group, and the occurrence of more than one IMID was not permitted in the IMID group at enrollment.

Patients provided written informed consent to participate in the study. The study protocol was reviewed and approved by the Health Research Ethics Board of the University of Manitoba.

The sample size for the present study was determined based on a power analysis for the two principal longitudinal study objectives. Details of the sample size calculation and comprehensive protocol information is available elsewhere [43].

### Measures

#### Demographics

A questionnaire was used to collect information about general demographic characteristics including age, sex, educational attainment, ethnicity (Caucasian or other), and occupation (according to National Occupational Classification)[44].

#### Work impairment

Work Productivity and Activity Impairment (WPAI) [45]. The WPAI is a 6 item self-report measure of work-related productivity losses and general activity impairment, with strong evidence of validity and reliability in various populations including individuals with IMIDs [29,46,47]. The time interval of assessment is the past 7 days. For the purposes of the present investigation, three aspects of activity impairment were used: a) percentage of work time missed due to health (absenteeism); b) percentage of impairment at work due to health (presenteeism); and c) percent of activity impairment due to health (general activity impairment). Measures of absenteeism and presenteeism were applicable to employed participants only, whereas the measure of general activity impairment was applicable to all participants. Each of these dependent variables was analyzed separately as it was considered probable that their determinants might differ.

#### Distress symptoms

MOS-Modified Pain Effects Scale (MOS-Pain): The Pain Effects Scale was originally developed and validated for the Medical Outcomes Study [48]. In the present version a reduced, validated 6-item version with scores ranging from 6 to 30 was utilized [49]. This version assesses the impact of pain and unpleasant sensations on function and well-being. The time interval of assessment is the past 4 weeks, with a higher score reflecting a higher impact of pain on functioning. Cronbach’s alpha for the MOS-Pain scale in the present study was 0.92.

Fatigue Impact Scale for Daily Use (D-FIS)[50]: The D-FIS, which was adapted from the original Fatigue Impact Scale [51], is a validated, 8-item self-report scale which rates the degree to which fatigue impacts on function, with a time interval of assessment of past day, and a higher score reflecting a higher impact of fatigue. It has good psychometric properties [52]. Higher scores have been associated with more hours lost from work and lower self-rated work productivity in persons experiencing flu-like symptoms [50]. Cronbach’s alpha for the D-FIS in the present study was 0.95.

Hospital Anxiety and Depression Scale (HADS)[53]. The HADS is a 14-item self-report measure that assesses depression symptoms (7 items) and anxiety symptoms (7 items). The time interval of assessment is the past week. The item content of the HADS minimizes symptomatic overlap with physical disorders and aims to distinguish carefully between manifestations of anxiety (HADS-A) and depression (HADS-D). The validity of the HADS has been demonstrated across multiple patient and general population samples [54]. In the present study Cronbach’s alpha was 0.88 for the HADS-A and 0.85 for the HADS-D.

#### Physical and cognitive variables

Self-Administered Comorbidity Questionnaire [55]. This self-report questionnaire is a validated instrument for recording concurrent health conditions in both general population and medically-ill samples [55,56]. The comorbid conditions considered included hypercholesterolemia, hypertension, heart disease, peripheral vascular disease, lung disease, diabetes, breast cancer, colon cancer, lung cancer, skin cancer, other cancer, migraine, thyroid disease, lupus, osteoarthritis, osteoporosis, fibromyalgia, kidney disease, peptic ulcer, liver disease, and epilepsy. For the present investigation, a tally of the number of concurrent conditions was scored as 0, 1, 2, or 3+ conditions to yield a measure of the total burden of comorbid conditions.

Physical function assessments included measures of lower limb and upper limb function. Participants completed the timed 25-second walk test (T25FW)[57,58], a reliable and valid performance measure for which established norms are available, and the Nine Hole Peg Test (9HPT) which has been validated in multiple medical illness populations including multiple sclerosis and rheumatoid arthritis [59,60]. Physical function z-scores were calculated by averaging the z-scores for each of the T25FW and 9HPT. The mean and standard deviation used in the z-scores were based on the entire cohort.

Cognitive function assessments included the Letter Number Sequencing test (LNS)[61], California Verbal Learning Test (CVLT)[62], and the oral version of the and Symbol Digit Modalities Test (SDMT)[63]. The LNS score was the total raw score ranging from 3 to 20. The SDMT score was the total raw score ranging from 5 to 103. The CVLT score was the raw score of item 5, ranging from 0 to 16. Raw scores rather than age/sex/education based standardized scores were used in the present regression analyses because the planned regression models all included age, sex and education as control variables.

#### Mental disorders

Mental disorder diagnoses were assessed with the use of the Structured Clinical Interview for DSM-IV Axis I Mental Disorders (SCID)[64]. Diagnostic assessment with the SCID was used to ensure a carefully diagnosed anxiety and depression group and for descriptive purposes. In the present study we report the prevalence of past-year Major Depressive Disorder (SCID Depression) or any past year anxiety disorder (SCID Anxiety).

For the purposes of evaluating the association of depression and anxiety with work and activity impairment in the present study, depression and anxiety symptoms were assessed with the HADS.

### Analyses

Descriptive analyses were conducted for all participants and for the subset of participants who were employed. In preliminary analyses, employed and not-working participants were compared using t-tests with pooled variance for variables described using a mean and standard deviation, Wilcoxon test for variables described using a median and chi-square tests for categorical variables. These analyses were restricted to participants 63 years of age or less, as 63 years is the median age of retirement in Canada [65]. An additional preliminary analysis included Pearson correlations among the four distress variables.

None of the dependent variables (percentage of work time missed due to health; percentage of activity impairment due to health; general activity impairment) were normally distributed. Further, we anticipated that the correlates of work and activity impairment may differ at the extremes of the distribution. Accordingly, the main analyses were conducted using quantile regression analyses [66]; the 50^th^ and 90^th^ percentiles were the focus of inference, and 95% confidence intervals (95% CIs) were reported. Quantile regression is used to model the relationship between a set of independent variables and selected percentiles of the distribution of the dependent variable, allowing a determination of how different percentile levels of the dependent variable may be more affected than others. Analyses were conducted for each of the four distress variables of interest (fatigue impact, pain effects, depression, and anxiety) and the impairment outcomes (percent of work time missed due to health; percent of impairment at work due to health; general activity impairment). All models included sociodemographic factors (age, sex, education, ethnicity, and occupation), group membership (IMID group versus mental health group), physical function and cognitive function. In one set of models, the four distress variables were entered individually (one distress variable per model), to consider the independent association of each distress variable with work and general activity impairment, after covariate adjustment. In a second set of models, the four distress variables were entered together, after covariate adjustment. Standardized coefficients (mean 0 and variance 1) were calculated for all of the distress variables in the quantile regression models, in order to judge the effect sizes of the significant associations. All statistical analyses were conducted using SAS/STAT® V9.4 (SAS Institute Inc., Cary, NC). Participants with missing data were excluded from analyses.

## Results

Descriptive information for the employed subsample (N = 497, used in analyses for percent of work time missed due to health and percent of impairment at work due to health) and total sample (N = 964, used in analyses for general activity impairment) is shown in Table 1.

**Table 1:** Baseline characteristics for the total sample and employed subsample, stratified by illness group (IMID versus anxiety/depression).

Variable	Total Sample			Employed Sub-Sample		
	Total	IMID	Anxiety/ Depression	Total	IMID	Anxiety/ Depression
N	964	656	308	497	318	179
Background						
Mean age (SD)	49.2 (14.2)	51.7 (14.1)	43.9 (12.9)	44.5 (12.0)	45.9 (12.0)	42.0 (11.5)
% Female	75.6	75.2	76.6	74.7	73.0	77.7
% Caucasian	90.0	90.7	88.6	92.5	92.5	92.7
% Education						
High school or less	32.9	32.8	33.1	22.5	22.0	23.5
College/technical	37.3	37.8	36.4	39.4	40.9	36.9
University	29.8	29.4	30.5	38.1	37.1	39.7
% Occupation						
Management and administration	34.1	35.1	32.1	35.2	35.2	35.2
Health	18.2	15.9	23.1	20.9	17.9	26.3
Education, law, social	20.3	21.3	18.2	20.7	21.1	20.1
Other	27.4	27.7	26.6	23.1	25.8	18.4
Physical & Cognitive Functioning						
Median Physical function z-score (Q1, Q3)	0.1 (-0.3, 0.5)	0.1 (-0.4, 0.4)	0.3 (-0.01, 0.5)	0.3 (0.03, 0.6)	0.3 (-0.01, 0.6)	0.3 (-0.1, 0.6)
Mean SDMT score (SD)	56.0 (12.8)	54.5 (12.7)	59.2 (12.2)	60.8 (11.4)	59.8 (11.3)	62.5 (11.5)
Mean LNS score (SD)	10.6 (2.7)	10.4 (2.7)	10.9 (2.8)	11.4 (2.5)	11.3 (2.5)	11.6 (2.5)
Median CVLT item 5 score (Q1, Q3)	13 (11, 15)	13 (11, 14)	14 (11, 15)	14 (12, 15)	13 (11, 15)	14 (12, 15)
% Comorbid Health Conditions						
0	27.1	27.3	26.6	33.0	35.2	29.0
1	28.2	28.5	27.6	28.4	29.9	25.7
2	18.6	19.4	16.9	19.3	19.5	19.0
3+	26.1	24.9	28.9	19.3	15.4	26.3
Distress Symptoms						
Pain Effects Median MOS (Q1, Q3)	13 (9, 18)	12 (8, 17)	15 (11, 21)	12 (8, 16)	11 (7, 15)	13 (10, 18)
Fatigue Impact Median D-FIS (Q1, Q3)	11 (4, 18)	9 (3, 16)	14 (7, 20)	9 (3, 16)	7 (2, 13)	12 (7, 19)
Anxiety Median HADS-A(Q1, Q3)	8 (4, 11)	6 (3, 9)	12 (9, 14)	7 (4, 11)	6 (3, 9)	11 (8, 14)
Depression Median HADS-D (Q1, Q3)	5 (2, 8)	4 (2, 7)	8 (5, 11)	4 (2, 8)	3 (1, 6)	7 (4, 10)
Mental Disorders SCID						
% Anxiety disorder	35.7	21.5	65.9	37.0	20.1	67.0
% Major Depression	53.1	39.8	81.5	53.9	38.4	81.6
Impairment-WPAI						
Absenteeism Median % (Q1, Q3)				0 (0, 7.7)	0 (0, 3.0)	0 (0, 20.8)
Presenteeism Median % (Q1, Q3)				20 (0, 40)	20 (0, 30)	30 (10, 50)
General activity impairment Median % (Q1, Q3)	40 (10, 70)	30 (10, 60)	60 (30, 70)	30 (10, 60)	20 (0, 50)	50 (20, 70)

Notes: IMID = immune-mediated inflammatory diseases group including multiple sclerosis, inflammatory bowel disease and rheumatoid arthritis. SDMT = Symbol Digit Modalities Test; LNS = Letter Number Sequencing Test; CVLT = California Verbal Learning Test; MOS = Medical Outcomes Survey; D-FIS = Fatigue Impact Scale for Daily Use; HADS = Hospital Anxiety and Depression Scale; SCID = Structured Clinical Interview for DSM-IV Mental Disorders; WPAI = Work Productivity and Activity Impairment scale

In preliminary analyses (shown in Table 2), employed participants differed from not-working participants in that they were younger, had more years of education, were more likely to work in management or health-related occupations, were less likely to have MS or RA, and had better physical and cognitive function (all comparisons p < 0.001). The employed subjects also had lower scores on measures of fatigue impact, pain effects, depression, and anxiety (all comparisons p < 0.001).

**Table 2:** Frequencies, means and medians of characteristics for the employed group compared to the not-working group.

Variable	Employed	Not-Working	p-value
N	497	332	
Background			
Mean age (SD)	44.5 (12.0)	47.7 (12.3)	0.0002
% Female	371 (74.6)	257 (77.4)	0.3633
% Caucasian	459 (92.5)	275 (83.6)	<0.0001
% Education			
High school or less	112 (22.5)	152 (45.8)	<0.0001
College/technical	196 (39.4)	115 (34.6)	
University	189 (38.1)	65 (19.6)	
% Occupation			
Management and administration	175 (35.2)	100 (30.1)	0.0010
Health	104 (20.9)	53 (16.0)	
Education, law, social	103 (20.7)	60 (18.1)	
Other	115 (23.1)	119 (35.8)	
Disease Group			
Inflammatory Bowel Disease	154 (31.0)	64 (19.3)	0.0003
Multiple Sclerosis	115 (23.1)	99 (29.8)	
Rheumatoid Arthritis	49 (9.9)	51 (15.4)	
Anxiety/Depression	179 (36.0)	118 (35.5)	
Physical & Cognitive Functioning			
Median physical function z-score (Q1, Q3)	0.3 (0.03, 0.6)	0.001 (-0.5, 0.4)	<0.0001
Mean SDMT score (SD)	60.8 (11.4)	53.0 (12.3)	<0.0001
Mean LNS score (SD)	11.4 (2.8)	9.9 (2.7)	<0.0001
Median CVLT item 5 score (Q1, Q3)	14 (12, 15)	13 (10, 14)	<0.0001
% Comorbid Health Conditions			
0	164 (33.0)	89 (26.8)	0.0563
1	141 (28.4)	97 (29.2)	
2	96 (19.3)	58 (17.5)	
3+	96 (19.3)	88 (26.5)	
Distress Symptoms			
Pain Effects Median MOS (Q1, Q3)	12 (8, 16)	16 (11, 21)	<0.0001
Fatigue Impact Median D-FIS (Q1, Q3)	9 (3, 16)	14 (7, 21)	<0.0001
Anxiety Median HADS-A (Q1, Q3)	7 (4, 11)	9 (5, 13)	0.0005
Depression Median HADS-D (Q1, Q3)	4 (2, 8)	7 (4, 10)	<0.0001
Mental Disorders SCID			
% Anxiety disorder	37.0	41.6	0.1884
% Major Depression	53.9	62.4	0.0163

Notes: The Not-Working group was restricted to participants ≤ 63 years of age (based on the median retirement age in Canada). T-tests with pooled variance were used for variables described using a mean and standard deviation. Wilcoxon test was used for variables described using a median. Chi-square tests were used for tests of proportion. SDMT = Symbol Digit Modalities Test; LNS = Letter Number Sequencing Test; CVLT = California Verbal Learning Test; MOS = Medical Outcomes Survey; D-FIS = Fatigue Impact Scale for Daily Use; HADS = Hospital Anxiety and Depression Scale; SCID = Structured Clinical Interview for DSM-IV Mental Disorders

Correlations among the distress measures and WPAI impairment are shown in Table 3. All correlations among HADS depression, HADS anxiety, MOS pain effects, and DFIS fatigue impact were statistically significant. The lowest of these correlations was between anxiety and pain (r = 0.48) and the highest of these correlations was between depression and fatigue (r = 0.66).

**Table 3:** Pearson correlations among distress measures and WPAI impairment.

Variable	Pain Effects MOS	Fatigue Impact DFIS	Anxiety HADS-A	Depression HADS-D	Absenteeism (N = 467)	Presenteeism (N = 471)
Fatigue Impact D-FIS	0.66	1				
Anxiety HADS-A	0.48	0.55	1			
Depression HADS-D	0.60	0.66	0.65	1		
Absenteeism (N = 467)	0.35	0.38	0.33	0.37	1	
Presenteeism (N = 471)	0.55	0.56	0.46	0.50	0.57	1
General Activity Impairment	0.66	0.70	0.48	0.62	0.42	0.70

Notes: The correlations for absenteeism and presenteeism are based on the employed sub-sample only. MOS = Medical Outcomes Survey; D-FIS = Fatigue Impact Scale for Daily Use; HADS = Hospital Anxiety and Depression Scale; WPAI = Work Productivity and Activity Impairment scale. All correlations are p < 0.0001.

The results of quantile regression analyses examining the individual association of four distress variables with work and general activity impairment are shown in Table 4. Each of pain effects, fatigue impact, anxiety and depression were significantly associated with absenteeism (percent of time missed due to health), presenteeism (percent of impairment at work due to health), and general activity impairment. The effect sizes were generally medium to large, except for the associations with general activity impairment at the 90^th^ percentile, which were small to medium sized.

**Table 4:** Quantile regression point estimates and standardized estimates with 95% confidence intervals for absenteeism (percent of work time missed due to health), presenteeism (percent of impairment at work due to health), and general activity impairment (percent of activity impairment due to health). Distress variables examined individually in separate regression models.

Variable	50^th^ percentile	90^th^ percentile
Absenteeism**		
MOS–pain effects	-	**2.6 (1.7, 3.5)**
MOS–pain effects*	*-*	***0*.*4 (0*.*2*, *0*.*6)***
DFIS–fatigue impact	-	**3.0 (2.4, 3.7)**
DFIS–fatigue impact*	*-*	***1*.*0 (0*.*8*, *1*.*2)***
HADS anxiety	-	**3.3 (1.9, 4.7)**
HADS anxiety*	*-*	***0*.*5 (0*.*3*, *0*.*8)***
HADS depression	-	**4.8 (3.8, 5.7)**
HADS depression*	*-*	***0*.*8 (0*.*6*, *0*.*9)***
**Presenteeism**		
MOS–pain effects	**3.1 (2.6, 3.5)**	**3.0 (2.1, 3.8)**
MOS–pain effects*	***0*.*7 (0*.*6*, *0*.*8)***	***0*.*6 (0*.*4*, *0*.*8)***
DFIS–fatigue impact	**2.1 (1.8, 2.4)**	**2.6 (2.1, 3.1)**
DFIS–fatigue impact*	***0*.*7 (0*.*6*, *0*.*8)***	***0*.*8 (0*.*6*, *1*.*0)***
HADS anxiety	**2.7 (2.2, 3.2)**	**2.6 (1.2, 4.0)**
HADS anxiety*	***0*.*5 (0*.*4*, *0*.*6)***	***0*.*5 (0*.*2*, *0*.*7)***
HADS depression	**3.6 (2.8, 4.4)**	**4.4 (2.9, 6.0)**
HADS depression*	***0*.*5 (0*.*4*, *0*.*7)***	***0*.*7 (0*.*4*, *0*.*9)***
**General Activity Impairment**		
MOS–pain effects	**3.8 (3.5, 4.0)**	**1.9 (1.4, 2.4)**
MOS–pain effects*	***0*.*8 (0*.*7*, *0*.*8)***	***0*.*4 (0*.*3*, *0*.*5)***
DFIS–fatigue impact	**2.7 (2.5, 2.9)**	**1.5 (1.1, 1.8)**
DFIS–fatigue impact*	***0*.*8 (0*.*7*, *0*.*8)***	***0*.*4 (0*.*3*, *0*.*5)***
HADS anxiety	**3.3 (2.6, 3.9)**	**1.7 (1.2, 2.1)**
HADS anxiety*	***0*.*5 (0*.*4*, *0*.*6)***	***0*.*3 (0*.*2*, *0*.*4)***
HADS depression	**4.4 (4.0, 4.8)**	**2.6 (1.9, 3.3)**
HADS depression*	***0*.*6 (0*.*6*, *0*.*7)***	***0*.*4 (0*.*3*, *0*.*5)***

Notes: Absenteeism, Presenteeism and General Activity Impairment were assessed for the past 7 days using the Work Productivity and Activity Impairment scale (WPAI). Values in bold font indicate estimates that were statistically significant at α = 0.05.

*Values in italics are the standardized estimates.

**The 50th percentile results for Absenteeism could not be calculated as 70% of the working subjects reported zero missed time in the past week.

Each regression analysis controlled for the following co-variates: age, sex, education, ethnicity, occupation, physical function, cognitive function, comorbidity, and disease group. MOS = Medical Outcomes Survey; D-FIS = Fatigue Impact Scale for Daily Use; HADS = Hospital Anxiety and Depression Scale.

The results of three models that examined the associations of four distress variables (entered together) with absenteeism, presenteeism, and general activity interference respectively are shown in Table 5. In each model, two or three of the distress variables were significant predictors of impairment. No noteworthy differences in the sizes of associations were observed for the 50th and 90th percentiles. Fatigue impact was a significant predictor of impairment in all of the analyses, with effect sizes in the small to medium range (standardized coefficients of 0.2 to 0.5). Among these four distress variables, anxiety appeared to have the least consistent association with impairment, with only one small but statistically significant association with impairment noted (presenteeism at the 50^th^ percentile level; standardized coefficient 0.2).

**Table 5:** Quantile regression point estimates 95% confidence intervals for absenteeism (percent of work time missed due to health) (N = 457), presenteeism (percent impairment while at work) (N = 462), and general activity impairment (N = 939).

Variable	Absenteeism**	Presenteeism		General Activity Impairment	
	90^th^ percentile	50^th^ percentile	90^th^ percentile	50^th^ percentile	90^th^ percentile
Intercept	19.8 (-19.1, 58.8)	15.9 (-3.2, 35.1)	25.4 (-25.3, 76.0)	-3.0 (-16.4, 10.5)	**48.9 (24.2, 73.5)**
Age	-0.1 (-0.5, 0.2)	**-0.2 (-0.4, -0.1)**	-0.3 (-0.7, 0.2)	**-0.1 (-0.2, -0.01)**	**-0.3 (-0.5, 0.04)**
Sex					
Female	1.1 (-6.5, 8.7)	-2.8 (-6.5, 0.9)	3.8 (-3.5, 11.2)	1.9 (-0.7, 4.5)	**7.0 (2.0, 12.1)**
Male	reference	reference	reference	reference	reference
Education					
High school or less	reference	reference	reference	reference	reference
College/technical	2.7 (-6.3, 11.5)	-3.0 (-7.3, 1.3)	0.4 (-8.8, 9.5)	2.1 (-0.6, 4.7)	1.4 (-3.6, 6.5)
University	-3.6 (-10.9, 3.7)	0.1 (-4.1, 4.3)	9.6 (-0.9, 20.1)	1.2 (-1.8, 4.2)	1.3 (-5.6, 8.2)
Ethnicity					
Caucasian	1.4 (-8.2, 11.2)	1.1 (-5.6, 7.8)	1.5 (-16.2, 19.2)	-3.8 (-8.3, 0.8)	-3.9 (-14.1, 6.3)
Other	reference	reference	reference	reference	reference
Occupation					
Education, law, social	1.5 (-8.2, 11.2)	2.0 (-2.9, 6.9)	8.7 (-5.4, 22.8)	-0.2 (-3.8, 3.3)	6.8 (-1.2, 14.9)
Health	6.0 (-6.1, 18.1)	1.3 (-4.7, 7.2)	4.2 (-8.0, 16.4)	1.1 (-2.5, 4.6)	-1.1 (-8.0, 5.9)
Management and administration	-0.8 (-9.2, 7.7)	-1.0 (-5.4, 3.3)	1.1 (-7.3, 9.5)	0.2 (-2.7, 3.2)	3.8 (-1.6, 9.2)
Other	reference	reference	reference	reference	reference
Physical function	**-4.5 (-8.8, -0.1)**	-0.3 (-4.0, 3.4)	5.5 (-1.1, 12.0)	**-3.0 (-4.7, -1.3)**	**-4.0 (-8.0, -0.1)**
SDMT score	-0.04 (-0.4, 0.3)	-0.1 (-0.3, 0.1)	-0.4 (-0.8, 0.03)	0.003 (-0.1, 0.1)	-0.1 (-0.4, 0.1)
LNS score	0.7 (-1.0, 2.3)	0.3 (-0.5, 1.1)	1.7 (-0.3, 3.7)	-0.3 (-0.8, 0.2)	-0.2 (-1.4, 1.0)
CVLT item 5 score	-0.9 (-2.5, 0.6)	**-0.8 (-1.6, -0.05)**	-1.1 (-2.5, 0.3)	0.1 (-0.3, 0.6)	0.4 (-0.6, 1.4)
Comorbidity					
0	reference	reference	reference	reference	reference
1	-0.7 (-7.0, 5.6)	1.9 (-1.9, 6.0)	-2.6 (-11.1, 5.8)	2.4 (-0.2, 5.1)	0.7 (-5.2, 6.5)
2	3.2 (-7.3, 13.8)	-0.1 (-5.2, 5.0)	9.3 (-2.2, 20.8)	-1.7 (-5.6, 2.1)	2.9 (-5.8, 11.6)
3+	2.9 (-10.4, 16.2)	0.8 (-4.7, 6.3)	-1.6 (-12.9, 9.6)	3.0 (-0.9, 6.9)	-0.5 (-7.7, 6.7)
Disease group					
IMID	-7.4 (-18.4, 3.7)	-4.1 (-8.5, 0.3)	-7.4 (-16.5, 1.8)	0.6 (-2.7, 3.9)	-5.0 (-10.8, 0.8)
Psych	reference	reference	reference	reference	reference
MOS–pain effects	0.2 (-0.6, 1.0)	**1.7 (1.0, 2.3)**	**1.0 (0.2, 1.8)**	**1.9 (1.5, 2.3)**	**1.2 (0.8, 1.5)**
MOS–pain effects*	*0*.*05 (-0*.*1*, *0*.*2)*	***0*.*4 (0*.*2*, *0*.*5)***	***0*.*2 (0*.*01*, *0*.*4)***	***0*.*4 (0*.*3*, *0*.*5)***	***0*.*2 (0*.*2*, *0*.*3)***
DFIS–fatigue impact	**1.6 (0.5, 2.6)**	**0.7 (0.2, 1.2)**	**1.6 (0.9, 2.3)**	**1.2 (0.9, 1.4)**	**1.1 (0.7, 1.4)**
DFIS–fatigue impact*	***0*.*5 (0*.*2*, *0*.*8)***	***0*.*2 (0*.*1*, *0*.*3)***	***0*.*5 (0*.*3*, *0*.*7)***	***0*.*3 (0*.*3*, *0*.*4)***	***0*.*3 (0*.*2*, *0*.*4)***
HADS anxiety	-0.1 (-1.3, 1.2)	**0.9 (0.3, 1.4)**	1.1 (-0.2, 2.5)	0.03 (-0.4, 0.5)	-0.6 (-1.2, 0.1)
HADS anxiety*	*-0*.*01 (-0*.*3*, *0*.*2)*	***0*.*2 (0*.*1*, *0*.*3)***	*0*.*2 (-0*.*01*, *0*.*4)*	*0*.*004 (-0*.*1*, *0*.*1)*	*-0*.*1 (-0*.*2*, *0*.*004)*
HADS depression	**3.2 (1.4, 5.0)**	0.6 (-0.3, 1.5)	1.4 (-0.01, 2.8)	**1.3 (0.8, 1.9)**	**1.9 (1.2, 2.6)**
HADS depression*	***0*.*5 (0*.*3*, *0*.*8)***	*0*.*1 (-0*.*04*, *0*.*2)*	*0*.*2 (-0*.*01*, *0*.*4)*	***0*.*2 (0*.*1*, *0*.*3)***	***0*.*3 (0*.*2*, *0*.*4)***

Notes: Absenteeism, Presenteeism and General Activity Impairment were assessed for the past 7 days using the Work Productivity and Activity Impairment scale (WPAI). Values in bold face font indicate estimates that were statistically significant at α = 0.05.

*Values in italics are the standardized estimates.

**The 50^th^ percentile results for Absenteeism could not be calculated as 70% of the employed participants reported zero missed time in the past week.

SDMT = Symbol Digit Modalities Test; LNS = Letter Number Sequencing Test; CVLT = California Verbal Learning Test; MOS = Medical Outcomes Survey; D-FIS = Fatigue Impact Scale for Daily Use; HADS = Hospital Anxiety and Depression Scale.

## Discussion

The results of the present study were in keeping with our expectation that several forms of distress symptoms would be associated with work and activity impairment in a moderately large chronic illness sample with diverse IMIDs and mental disorders. Notably, fatigue impact, pain effects, depression symptoms and anxiety symptoms were each associated with percentage of work time missed (absenteeism), percent of impairment while at work (presenteeism) and general functional impairment even after accounting for the effects of demographic differences, physical impairment, cognitive function and other concurrent medical problems. Not-working participants in comparison to employed participants endorsed higher levels of each form of distress.

There are several striking aspects of these findings. First, when the four distress variables were considered separately, they were each consistently associated with work and activity impairment regardless which scale of the WPAI was used in the analyses. We purposefully utilized a functional measure that assesses various aspects of impairment, anticipating that there might be differences in the determinants of these different aspects. However, each of pain effects, fatigue impact, depression and anxiety showed significant associations with each of absenteeism, presenteeism and general activity impairment. Second, we used a quantile regression analytic method in anticipation that more extreme levels of disability (ie 90^th^ percentile of impairment) might have more robust and striking associations with the distress variables. However we observed generally similar effects in the quantile regressions at the 50^th^ percentile as in the quantile regressions at the 90^th^ percentile, suggesting that the effects of the other three distress variables are not confined to those with the highest levels of disability. Third, in the multivariate analyses which included all four distress variables concurrently, the single distress variable that retained a significant association with work and activity impairment, for both of the percentile levels and for all three measured aspects of impairment, was fatigue impact. In other words, even when accounting for pain effects, depression and anxiety, fatigue impact remained associated with ability to attend work, ability to perform optimally at work, and to function outside of work, with small to medium sized effects (0.2 to 0.5). The selection of the fatigue impact scale for the present study (rather than a purely symptom-focused fatigue measure) could have influenced this outcome, as the “impact” of fatigue may align more directly with activity impairment than the symptom of fatigue.

In previous work, fatigue has been identified as a significant determinant of people with MS and RA leaving employment [67,68]. Interestingly, in studies of major depressive disorder, fatigue has also been identified as a correlate of work disability [69] and a predictor of poorer functional outcomes for pharmacological treatment [70]. As such, fatigue may be a particularly salient symptom to be addressed in a number of areas of clinical practice. Nevertheless, when considered individually, the other three distress variables (pain effects, depression and anxiety) each had significant associations with some aspect of work and/or general activity impairment. The association of depression with absenteeism, and the association of pain effects with general activity impairment (50^th^ percentile), as examples, showed large effect sizes (0.8). Because anxiety and depression were assessed using the HADS, a rating scale that aims to minimize symptoms that may reflect direct manifestations of physical illness, the observed associations between depression/anxiety and work impairment are presumed to be related to psychological symptoms.

In the combined analyses (Table 5) there were relatively few significant associations between the covariates (sociodemographic, physical function, and cognitive function) and work disability. The variable distinguishing between the IMID groups and the depression and anxiety group was not significant in any of the regression analyses. This suggests that distress symptoms, regardless whether they arise in the context of an IMID or a primary mental health diagnosis, contribute to work and activity impairment. Nevertheless, the comparisons of employed and not working participants demonstrated that individuals with MS and RA were less likely to be currently employed. Noteworthy associations in the combined analyses included an effect of physical function on both work absenteeism and general activity impairment, but not presenteeism. Intuitively, individuals who have physical impairments may experience hardship in getting to work or other meaningful activities. However, if they are able to overcome these obstacles and attend work, their physical limitations may not interfere with their work function, particularly if they are not participating in work that requires a high level of physical mobility or dexterity. The California Verbal Learning Task (item 5 score) was associated with work interference, but not absenteeism or general activity impairment. Reasonably, one might expect that verbal learning and memory deficits could reduce work productivity in many types of work, but may not directly interfere with attending work or other activities.

Fatigue, pain and concurrent mental health symptoms (depression and anxiety) are important therapeutic targets across a range of chronic diseases including IMIDs simply based on the distress and suffering that they cause. Because of the consistent association between these distressing symptoms and work and general activity impairment, therapeutic interventions directed at these symptoms also may be effective in improving functional status. The effect sizes observed suggest the possibility that clinical interventions targeting these distress variables have the potential to substantially impact on work and activity impairment. There has been increasing research and clinical attention to fatigue, pain, depression and anxiety in IMIDs, as reflected by the numerous recent publications and reviews on the effectiveness of psychosocial and biomedical interventions for these important disease manifestations in MS [71–73], RA [14,74,75] and IBD [76–78]. At the present time, however, much of the literature is characterized by modest quality evidence, small sample sizes, lack of replication, challenges with the selection of control groups, and limited follow-up durations [72,74,78].

The findings of the present study need to be considered in light of its strengths and limitations. The use of a standardized measure of function, the WPAI, allowed for evaluation of several aspects of work and activity impairment among participants who are able to work (rather than only comparing working versus disabled individuals). The collective sample size for the study was relatively large (over 450 employed participants and over 900 in the total sample), though the size of the individual disease groups was too small to permit analysis by individual illness groups. Ulcerative colitis and Crohn’s disease were considered together in the IBD group, so the study is unable to address questions related to differential impact of the distress symptoms in these two IBDs. However, the aim of the study was to identify a common set of factors across illness groups. The study was conducted using a cross-sectional design, so that inferences about causality or prediction cannot be made. In the absence of a clear theoretical rationale to prioritize and test specific interactions among independent variables, the present studied focused exclusively on main effects, rather than interactions. The study did not incorporate measures of disease activity for the three IMIDs. This reflects the challenges of working across diseases which use very different measures with different properties, and a common underlying metric has not yet been established. Nonetheless, we accounted for physical function and cognitive status (which were measured consistently across groups) which partially reflect disease status, particularly for MS and RA. The measures of distress symptoms and work impairment used in the present study assess varying time frames ranging from the past day (D-FIS) to the past 4 weeks (MOS Pain). These differences in time frame may have reduced the observed associations among the measures, and future studies should aim to align the time frame of the measures used to obviate this issue. Finally, it should be noted that the measure of depression and anxiety (HADS) is a symptom-based measure whereas the fatigue (D-FIS) and pain (MOS-pain) measures are impact scales. This difference in scale focus may have affected the findings.

In summary, fatigue impact, pain effects, depression symptoms and anxiety symptoms are associated with work and general activity impairment across several IMIDs. Effective interventions focused on these symptoms could enhance patient-oriented care and potentially reduce the burden of chronic disease. Further controlled studies of pharmacologic and non-pharmacologic treatment interventions, and measurement of functional outcomes in clinical trials are needed to inform clinical practice. Additional work is needed on interventions such as workplace accommodation [79] to support continued engagement in work among those with chronic health conditions.
